# A New Rat Model of Epileptic Spasms Based on Methylazoxymethanol-Induced Malformations of Cortical Development

**DOI:** 10.3389/fneur.2017.00271

**Published:** 2017-06-12

**Authors:** Eun-Hee Kim, Mi-Sun Yum, Minyoung Lee, Eun-Jin Kim, Woo-Hyun Shim, Tae-Sung Ko

**Affiliations:** ^1^Department of Pediatrics, CHA Gangnam Medical Center, CHA University, Seoul, South Korea; ^2^Department of Pediatrics, Asan Medical Center Children’s Hospital, University of Ulsan College of Medicine, Seoul, South Korea; ^3^Department of Radiology, Asan Medical Center, University of Ulsan College of Medicine, Seoul, South Korea

**Keywords:** methylazoxymethanol acetate, malformations of cortical development, epileptic spasms, GABA, fast oscillations

## Abstract

Malformations of cortical development (MCDs) can cause medically intractable epilepsies and cognitive disabilities in children. We developed a new model of MCD-associated epileptic spasms by treating rats prenatally with methylazoxymethanol acetate (MAM) to induce cortical malformations and postnatally with *N*-methyl-d-aspartate (NMDA) to induce spasms. To produce cortical malformations to infant rats, two dosages of MAM (15 mg/kg, intraperitoneally) were injected to pregnant rats at gestational day 15. In prenatally MAM-exposed rats and the controls, spasms were triggered by single (6 mg/kg on postnatal day 12 (P12) or 10 mg/kg on P13 or 15 mg/kg on P15) or multiple doses (P12, P13, and P15) of NMDA. In prenatally MAM-exposed rats with single NMDA-provoked spasms at P15, we obtain the intracranial electroencephalography and examine the pretreatment response to adrenocorticotropic hormone (ACTH) or vigabatrin. Rat pups prenatally exposed to MAM exhibited a significantly greater number of spasms in response to single and multiple postnatal NMDA doses than vehicle-exposed controls. Vigabatrin treatment prior to a single NMDA dose on P15 significantly suppressed spasms in MAM group rats (*p* < 0.05), while ACTH did not. The MAM group also showed significantly higher fast oscillation (25–100 Hz) power during NMDA-induced spasms than controls (*p* = 0.047). This new model of MCD-based epileptic spasms with corresponding features of human spasms will be valuable for future research of the developmental epilepsy.

## Introduction

Malformations of cortical development (MCDs) often lead to developmental delay, intellectual disabilities, and intractable epilepsy (1). Both environmental injuries and genetic mutations can disrupt early brain development, giving rise to MCDs (2). Depending on MCDs severity and pattern, epilepsies may manifest at almost any developmental stage from newborns to adults and some patients with MCDs present with epileptic spasms (3, 4). Several animal models have been generated to reproduce the pathologic characteristics of human MCDs for the investigation of the causal relationship between specific dysplastic lesions and functional abnormalities (5, 6). One such model is prenatal methylazoxymethanol (MAM)-induced MCDs. The DNA alkylating agent MAM is administered to pregnant rats during the fetal period of intense neurogenesis to disrupt cell proliferation and migration of both neocortical and hippocampal pyramidal neurons (7, 8), and these abnormalities closely resemble those observed in patients with MCDs (9). Despite the vast evidence for hyper-excitability and decreased seizure threshold in malformed brain regions (7, 10–12), there is little evidence for spontaneous epilepsy in this MCD model (5, 13).

Malformations of cortical development are a major cause of catastrophic epilepsies such as epileptic spasms (3, 4, 14) and symptomatic epileptic spasms with MCDs have worse prognosis than those with cryptogenic or idiopathic etiologies (15). Epileptic spasms associated with MCDs are characterized by the coexistence of focal seizures and spasms as well as transition to refractory epilepsy, including Lennox–Gastaut syndrome (14). Thus, effective control of spasms during a critical period is believed to result in better developmental outcomes (16). Although there is no consensus regarding optimal treatment for epileptic spasms, the most commonly used medical therapies are adrenocorticotropic hormone (ACTH) and vigabatrin. ACTH is generally thought to offer an added advantage over vigabatrin particularly in cases with genetic or unknown etiologies, whereas vigabatrin has been shown to be effective in the short-term treatment in the patients with tuberous sclerosis complex and epileptic spasms (17).

To discover effective therapeutic strategies based on the pathomechanism in this intractable epilepsy, appropriate animal model of epileptic spasms is a prerequisite. However, currently available animal models of the epileptic spasms are mostly genetic models or models of cryptogenic or postnatal toxic etiologies (18), and there are no published data on the acute model of epileptic spasms associated with MCDs. We have developed a new infant rat model of epileptic spasms by combining prenatal MAM exposure (7, 8) and postnatal *N*-methyl-d-aspartic acid (NMDA) injection to induce age-specific spasms (19). We validated the anatomical and behavioral alterations in young rats with prenatal MAM exposure and the susceptibility to NMDA-induced spasms during infancy. To test the resemblance to human MCDs-associated spasms, the response to currently available AEDs was also evaluated. In addition, electrophysiological patterns during NMDA-induced spasms were examined to investigate the underlying neurophysiological abnormalities leading to enhanced seizure susceptibility in this model.

## Materials and Methods

### Animals

**Figure 1:**
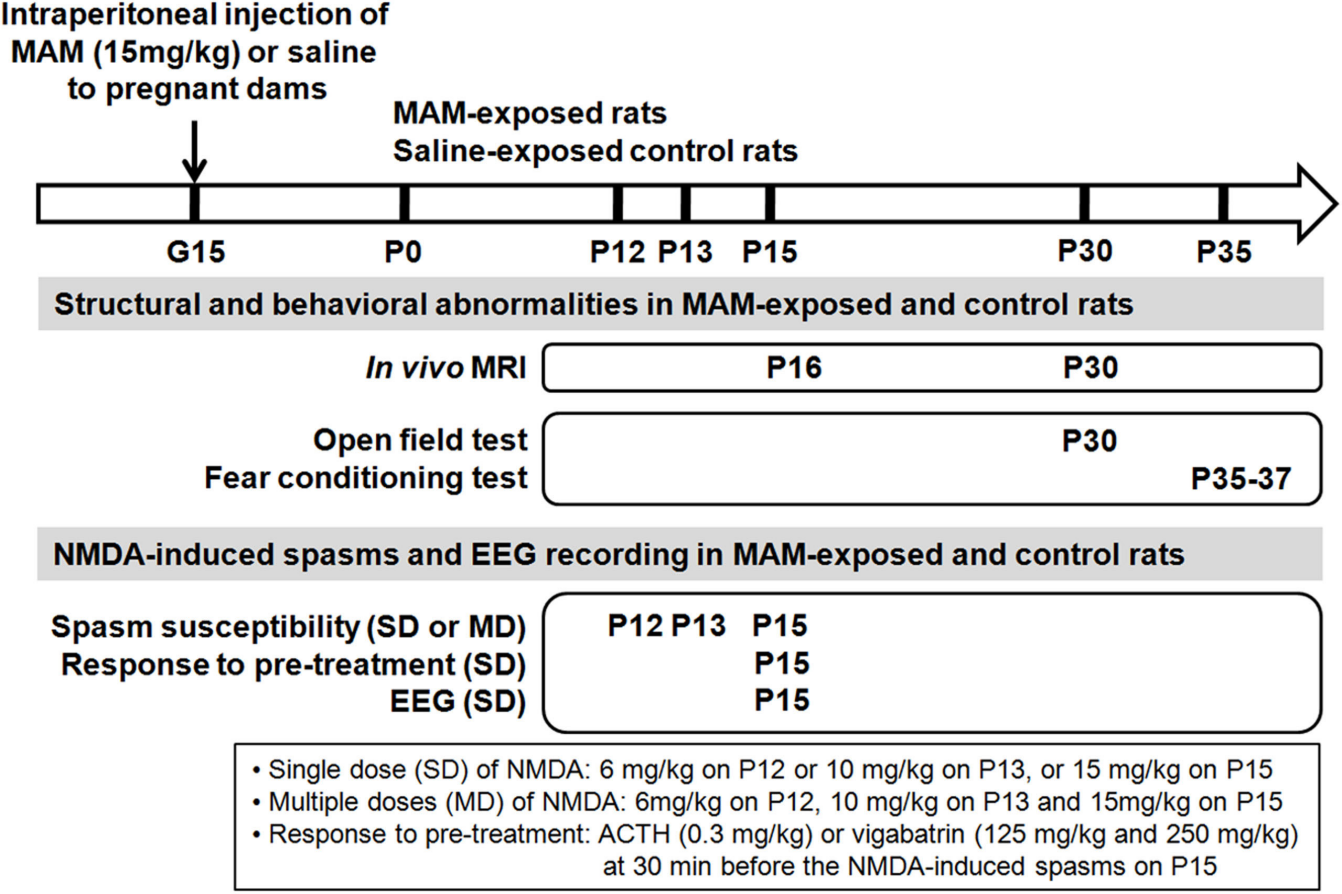
Schematic depiction of design of experiment. To produce cortical malformations to infant rats, methylazoxymethanol acetate (MAM), or saline vehicle were injected into pregnant dams at gestational day 15 (G15). The structural changes of the prenatally MAM-exposed rat brain were validated using *in vivo* magnetic resonance imaging (MRI) during infancy and juvenile period. Behavioral experiments were performed in MAM-exposed and the control during juvenile period. Spasms were triggered by single (SD) or multiple (MD) postnatal *N*-methyl-d-aspartate (NMDA) doses at infancy period in prenatally MAM-exposed rats and the controls. Spasms susceptibility, pre-treatment response to ACTH or vigabatrin, and the intractranial electroencephalography (EEG) were examined. P, postnatal day.

All experiments were approved by the Institutional Animal Care and Use Committee of the Ulsan University College of Medicine and conducted in accordance with the Revised Guide for the Care and Use of Laboratory Animals [NIH GUIDE, 25(28), 1996]. Fourteen pregnant Sprague-Dawley dams (Orient Bio, Seongnam, Korea) were acquired on gestational day (G) 13 and housed individually in the animal facility during the remainder of their pregnancy under a 12-h light/dark cycle and aseptic conditions with free access to food and water. To produce MCDs, eight dams were administered two doses of 15 mg/kg MAM (MRIglobal, Missouri) in 10 mL/kg saline at 0900 and 1800 hours on G15 by intraperitoneal (IP) injection. Six control pregnant rats were injected with saline vehicle. Delivery occurred consistently on G23, which was considered postnatal day (P) 0 for the offspring. Schematic depiction of experimental design and the rats used in each experiment are described in Figure 1 and Table 1.

**Table 1:** Summary of number of rats used in each experimental procedure.

Experimental group	MRI	Behavior study	EEG	Spasms susceptibility	
				Single dose	Multiple doses
Saline-exposed rats (*n* = 50)					
Saline injection (*n* = 16)	4	16	10	23	10 (1^a^)
NMDA injection (*n* = 34)					
MAM-exposed rats (*n* = 89)					
Saline injection (*n* = 14)	5	14	13 (1^a^)	62 (4^b^)	15
NMDA injection (*n* = 75)					

*MRI, magnetic resonance imaging; EEG, electroencephalography; MAM, methylazoxymethanol acetate; NMDA, N-methyl-d-aspartate*.

*^a^Indicates the number of mortality case during the NMDA-induced spasms*.

*^b^Indicates the death associated with high dose vigabatrin and spasms*.

### *In Vivo* Proton Magnetic Resonance Imaging (MRI)

**Figure 2:**
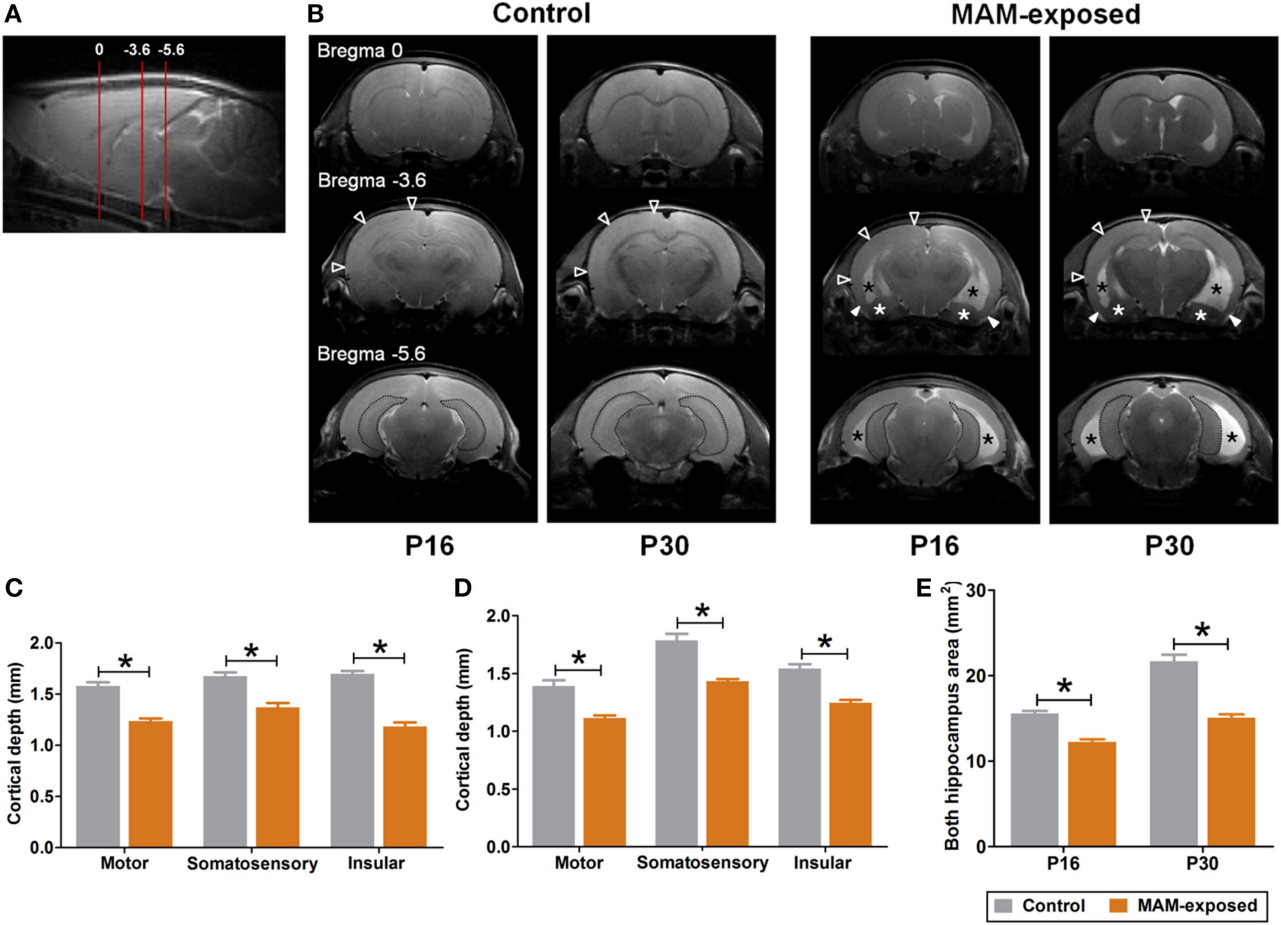
Representative T2-weighted magnetic resonance images and comparisons of cortical depth and hippocampal size between prenatally methylazoxymethanol acetate (MAM)-exposed rats and controls on postnatal day (P) 16 and P30. **(A)** Relative magnetic image locations (red lines) where cortical depth (3.5 mm posterior from the bregma) and hippocampal size (5.6 mm posterior from the bregma) were measured. **(B)** Compared to controls, MAM-exposed rats showed significant thinning of entorhinal cortex (filled arrowheads) and enlarged ventricles (black asterisks). Volume reduction and dysplasia were observed in both hippocampi (dotted area) and amygdala (white asterisks) of MAM-exposed rats at P16 and P30. To determine regional changes in cortical thickness, the depths of the motor, somatosensory, and insular cortices in one coronal section (empty arrowheads at bregma, −3.6 mm AP) were measured on P16 **(C)** and P30 **(D)**. Compared to controls (*n* = 4), the mean depths of motor (*p* = 0.016 at P16, *p* = 0.016 at P30), somatosensory (*p* = 0.016 at P16, *p* = 0.016 at P30), and insular (*p* = 0.016 at P16, *p* = 0.016 at P30) cortices were significantly decreased in MAM-exposed rats (*n* = 5). **(E)** A significant reduction in the mean area of both hippocampi (dotted area at the bregma, −5.6 mm) was also observed in MAM-exposed rats on P16 (*p* = 0.016) and P30 (*p* = 0.016). Values expressed as mean ± SEM. *p < 0.05 using the Mann–Whitney *U* test for comparison between the groups.

All proton (hydrogen-1 [^1^H])-MRI studies were conducted using a 9.4 T/160 mm Agilent animal MRI scanner (Agilent Technologies, Santa Clara, CA, USA) with 400 mT/m gradient system and a 38 mm 4-channel quadrature phased array coil for reception and 72 mm volume coil for transmission. During scanning, all animals were anesthetized by inhalation of 1.0% isoflurane in a 1:2 mixture of O_2_:N_2_O, and vital signs were monitored using a small-animal physiological monitoring device. ^1^H-MR images were acquired at P16 and P30 in the control (*n* = 4, 3 males and 1 female) and MAM-exposed rats (*n* = 5, 3 males and 2 females). T2-weighted images were acquired using a gradient echo sequence with the following imaging parameters: repetition time/echo time = 79/2.7 ms; flip-angle = 30°; 1 average; slice thickness (TH) = 2 mm; no inter-slice gap; field-of-view = 65 mm × 65 mm; matrix size = 128 × 128; receiver bandwidth = 50 kHz. To assess changes in cortical TH and hippocampal size in prenatally MAM-exposed and saline-exposed control rats, MRI data were analyzed using in-house software (AsanJ, Asan Medical Center, Seoul, Korea). Approximate anatomical positions were referenced relative to the bregma. The depths of the motor, somatosensory, and insular cortices in one coronal section (3.5 mm posterior from the bregma) and the area of both hippocampi in one coronal section (5.6 mm posterior from the bregma) were measured (Figure 2A).

### Behavioral Testing

Behavioral experiments were performed in the control (*n* = 16, 10 males and 6 females) and MAM-exposed rats (*n* = 16, 9 males and 7 females) at P30 and P35–37. The rats were separated from their mother at P23. Each experiment was conducted in a standard behavioral testing room during the light phase (0800–2000 hours) of the 12-h light–dark cycle.

#### Open Field Test

On P30, subjects were placed in an open field apparatus consisted of a black plastic box (60 cm × 60 cm with walls 30 cm high) and spontaneous locomotive activity was recorded for 5 min using a ceiling-mounted video camera. Each animal was tested only once and the arena was cleaned with 70% alcohol between tests. The motor and resting activities in the periphery (15 cm from each wall) and central zones were automatically analyzed using motion-tracking software (SMART 3.0, Panlab. S.L.U., Spain). Total distance traveled and times spent in central and peripheral zones were determined.

#### Fear Conditioning

Fear conditioning procedures were performed using an observation chamber (25 cm × 25 cm × 25 cm, Panlab S.L.U., Barcelona, Spain) constructed of aluminum (two side walls and ceiling) and Plexiglas (rear wall and hinged front door) situated in a soundproof box. The floor of the chamber consisted of 19 stainless steel rods (4 mm diameter) spaced 1.6 cm apart (center to center) connected to a shock generator and grid scrambler (Panlab S.L.U.) to deliver electrical footshocks. Both shock and conditions stimuli (CSs, tone and light) deliveries were controlled by a computerized system and rat behavior was recorded and analyzed using the signal generated by a high-sensitivity weight transducer system (Panlab S.L.U). The chambers were cleaned with 70% alcohol between tests.

On P35, tone (cued) conditioning was performed as described previously (20). Conditioning consisted of a 2 min habituation period in the chamber followed by exposure to five pairings of a tone (CS; 30 s, 85 dB, 2,000 Hz) with light illumination ending with a footshock (0.8 mA, 2.0 s) and a 20 s silent interval. Contextual fear memory formation was evaluated by scoring freezing behavior (defined as complete immobility except respiration) for 5 min in the absence of the tone and foot shock at 24 h after the conditioning trial (P36). Cued fear responses to the CS were measured on P37. To reduce the influence of context, tactile, and visual cues were manipulated. Following a 2 min period with no CS, the rats were presented with five tones (85 dB, 2 kHz, 30 s; 20 s between tone presentations) in the absence of foot shock, and freezing activity was quantified.

### Spasms Monitoring and Electroencephalography (EEG) Recording

In prenatally MAM- (*n* = 33, 17 males and 16 females) or saline- (*n* = 23, 12 males and 11 females) exposed rat pups, spasms were trigger by single dose (SD, 6 mg/kg on P12, 10 mg/kg on P13, or 15 mg/kg IP on P15, volume always 10 mL/kg in saline) or multiple doses (MD; P12, P13, and P15) of NMDA (Sigma, St. Louis, MO, USA) based on previous experiments (19, 21). The latency to onset of spasms (tail twisting and full spasms) and the total number of full spasms were recorded. The rats were monitored for 90 min as our pilot experiments demonstrated that spasms would disappear within 90 min.

To evaluate the acute pretreatment effect of the current drug the prenatally MAM-exposed rats with spasms triggered by single NMDA dose at P15 were pretreated with ACTH (*n* = 6, 6 females, synthetic human ACTH; Abcam Biochemicals, Bristol, UK; 0.3 mg/kg in 10 mL/kg saline IP) or vigabatrin [VGB; Abcam Biochemicals, Bristol, UK; 125 mg/kg (*n* = 11, 5 males and 6 females) or 250 mg/kg (*n* = 8, 2 males and 6 females) in 10 mL/kg saline IP] at 30 min before the injection of NMDA according to the acute administration paradigm (19) and the total number and the latencies to onset of full spasms were compared with those of MAM-SD rats with saline vehicle (*n* = 10, 3 males and 7 females).

Twelve MAM-exposed rat pups (7 males and 5 females) and ten saline-exposed controls (4 males and 6 females) were used for intracranial EEG. For EEG recordings, electrodes were surgically implanted in P13 pups while under sedation with ketamine/xylazine (50/7 mg/kg in 10 mL/kg saline IP). Two silver recording electrodes were implanted over both parietal cortices, while reference and ground lead screws were positioned above the cerebellum and near the bregma, respectively. The electrodes were connected to a multiple socket and secured to the skull with dental acrylic. At P15, EEGs of the two groups of rats were monitored with simultaneous and synchronized video using the Twin EEG system (Grass Technologies) for 1 h before NMDA injection and 2 h after injection or until the end of spasms. The sampling rate was 400 Hz and signals were bandpass filtered at 0.1–100 Hz. The intensity and the frequency distribution of the EEG were obtained from the power spectrum by fast Fourier Transform with a block size of 256. The absolute power (μV^2^) of each EEG frequency band [delta and theta (0.5–7 Hz), alpha (8–12 Hz), beta (13–24 Hz), and fast oscillations (FOs) (25–100 Hz)] at selected pre-NMDA resting periods (background activity) and during post-NMDA spasms and inter-spasms periods were recorded and compared between MAM and control rats. Color density spectral arrays (CDSAs) are used to display the FOs. Each CDSA trace contains 30 s of condensed EEG data from 25 to 100 Hz on the *y*-axis with color scale for power.

### Statistical Analyses

All data were analyzed using IBM SPSS (ver. 22.0; IBM Corp., Armonk, NY, USA). Group differences were assessed using the Kruskal–Wallis test, the Mann–Whitney *U* test, or independent *t*-test and appropriate Bonferroni correction was applied for multiple pair-wise comparisons to maintain an alpha level of *p* < 0.05. Repeated-measure ANOVA was used to analyze the effects of group and age on spasm susceptibility. In spectral analysis of EEG data (power vs. frequency), the random effect model was used to adjust for heterogeneity among EEG power values for each subject. A *p* value <0.05 was considered statistically significant.

## Results

### *In Vivo* Structural Abnormalities in MAM-Exposed Rats during Infancy and Juvenile Period

Figure 2 presents representative T2-weighted MR images of control (*n* = 4) and MAM-exposed rats (*n* = 5) acquired on P16 and P30. Compared to the normal brain of controls, MAM-exposed rats exhibited widespread cortical thinning, enlargement of lateral and third ventricles, and volume reduction and dysplasia of bilateral hippocampi and amygdalas at both P16 and P30 (Figure 2B). MAM group rats exhibited reduced mean TH of motor cortex (P16, control vs. MAM; 1.6 ± 0.03 vs. 1.2 ± 0.02 mm; P30, 1.4 ± 0.05 vs. 1.1 ± 0.02 mm; *p* = 0.016 for both), somatosensory cortex (P16, 1.7 ± 0.04 vs. 1.4 ± 0.04 mm; P30, 1.8 ± 0.06 vs. 1.4 ± 0.02 mm; *p* = 0.016 for both), and insular cortex (P16, 1.7 ± 0.03 vs. 1.2 ± 0.04 mm; P30, 1.5 ± 0.04 vs. 1.2 ± 0.02 mm; *p* = 0.016 for both) (Figures 2C,D). Compared to controls, the mean areas of bilateral hippocampi were also significantly reduced in MAM-exposed rats at both P16 and P30 (P16, 15.6 ± 0.3 vs. 12.2 ± 0.3 mm^2^; P30, 21.7 ± 0.8 vs. 15.1 ± 0.4 mm^2^; *p* = 0.016 for both) (Figure 2E).

### Behavioral Abnormalities in MAM-Exposed Young Rats

**Figure 3:**
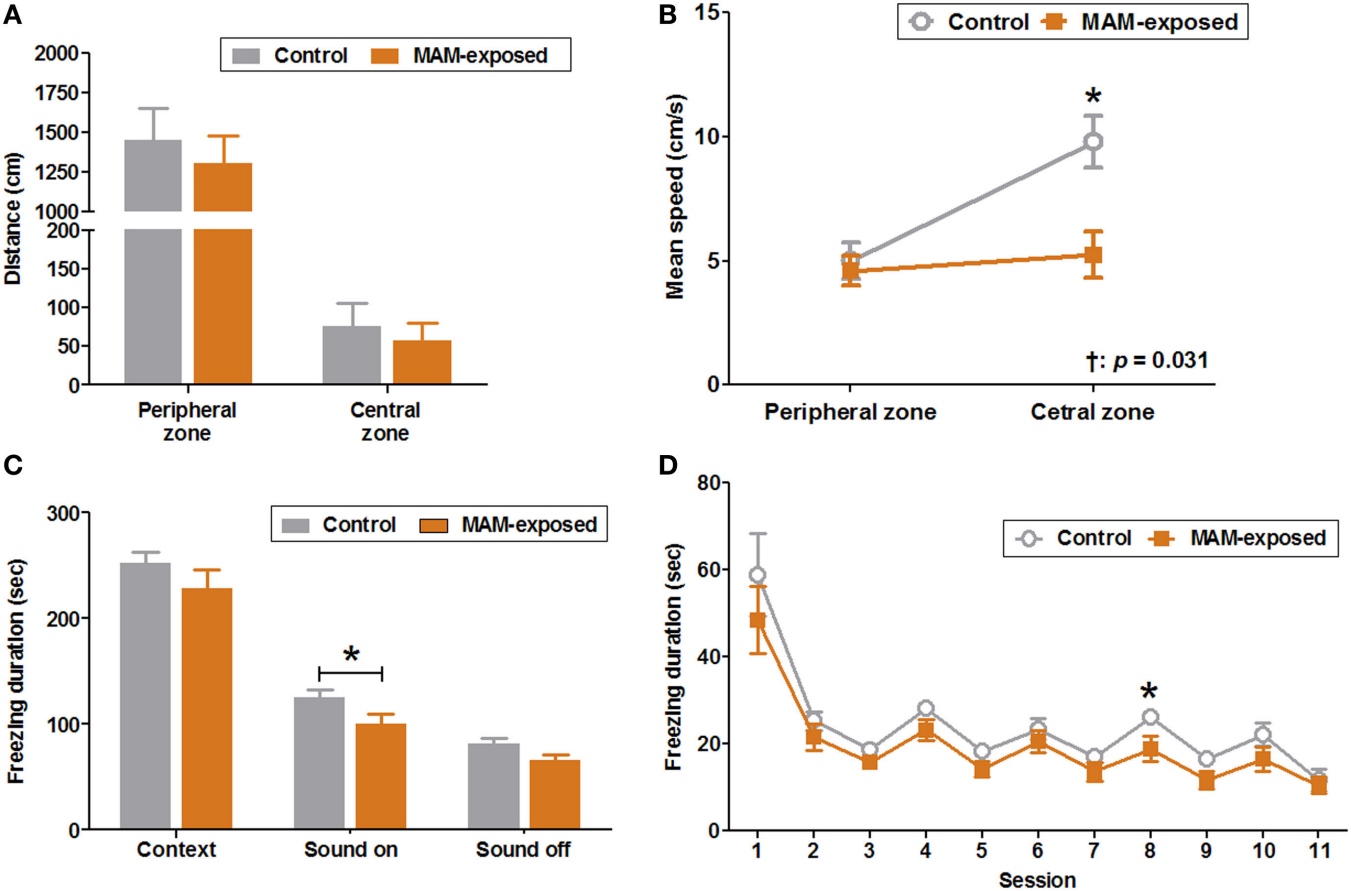
Behavioral abnormalities in prenatally methylazoxymethanol acetate (MAM)-exposed young rats. The open field test was performed at postnatal day (P) 30 and fear conditioning tests at P35–37. **(A)** The mean distances traveled in the peripheral and central zones did not differ between the groups. **(B)** Compared to controls (*n* = 16), MAM-exposed rats (*n* = 14) showed significantly slower mean speed in the central zone (9.8 ± 1.1 vs. 5.2 ± 0.9 cm/s, *p* = 0.016). The change in mean speed between peripheral and central zones differed significantly by group (*p* = 0.031). **(C)** The freezing response to context at P36 did not differ significantly between the groups. At P37, MAM-exposed rats showed significantly shorter freezing responses to conditioned sound stimuli (freezing duration with sound ON) (*n* = 16, 100.5 ± 9.6 s) than controls (*n* = 12, 124.8 ± 7.7 s, *p* = 0.047). **(D)** The freezing duration for each session in MAM-exposed rats and controls. The freezing duration on session 8 with a sound cue was significantly shorter for MAM-exposed rats (18.8 ± 3.0 s) than for controls (26.0 ± 1.2 s, *p* = 0.042). Values expressed as mean ± SEM. *p < 0.05 using the Mann–Whitney *U* test for comparison between the groups. ^†^*p* < 0.05 using the Mann–Whitney *U* test for comparison of speed between peripheral and central zones by group.

Results of the open field test at P30 for MAM-exposed rats (*n* = 14) and saline-exposed controls (*n* = 16) are presented in Figures 3A,B. The mean ambulation distances in peripheral and central zones did not differ significantly between groups (Figure 3A), but the mean speed in the central zone was significantly slower in the MAM group compared to controls (5.2 ± 0.9 vs. 9.8 ± 1.1 cm/s, *p* = 0.016) (Figure 3B).

The duration of freezing during conditioning on P35 and the freezing response to context on P36 did not differ significantly between groups. However, the freezing response to conditioned sound stimuli (freezing during sound ON) was significantly shorter in MAM-exposed rats than controls (100.5 ± 9.6 vs. 124.8 ± 7.7 s, *n* = 16 and 12, respectively, *p* = 0.047) with significantly reduced freezing duration to sound cue on the eighth session of P37 (18.8 ± 3.0 vs. 26.0 ± 1.2 s, *p* = 0.042) (Figures 3C,D).

### Elevated NMDA-Induced Spasm Susceptibility in MAM-Exposed Infant Rats

**Figure 4:**
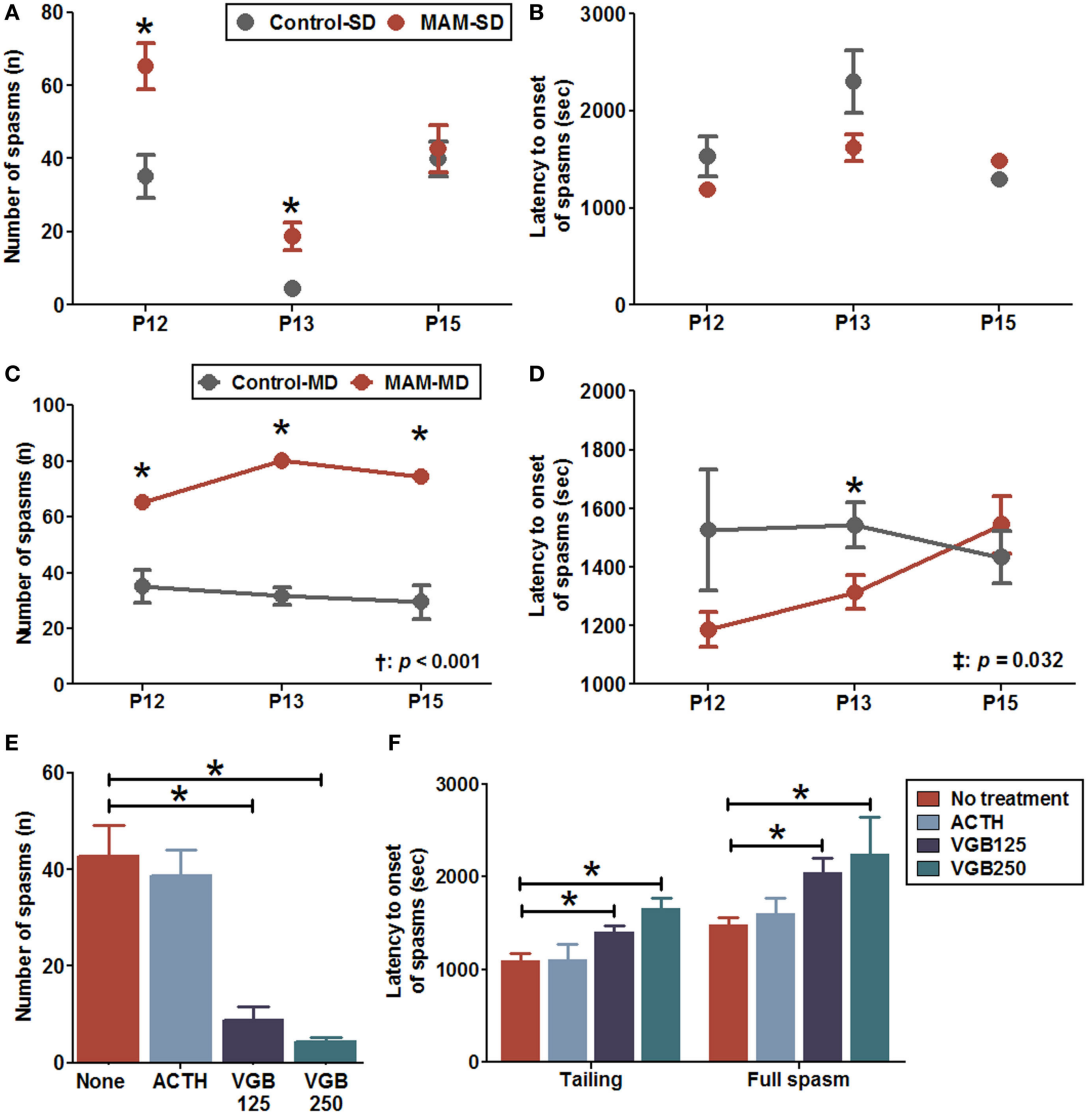
Enhanced NMDA-induced spasm susceptibility and effects of anti-epileptic drugs in prenatally MAM-exposed infant rats treated with single (SD) or multiple (MD) *N*-methyl-d-aspartate (NMDA) doses. **(A)** MAM-exposed rats (MAM-SD, *n* = 15 at postnatal day (P) 12, *n* = 8 at P13, *n* = 10 at P15) showed significantly increased numbers of spasms in response to single NMDA doses at P12 (65.3 ± 6.2 vs. 35.1 ± 5.9, *p* = 0.003) and P13 (18.8 ± 3.8 vs. 4.4 ± 1.2, *p* = 0.002) compared to prenatally saline-exposed rats (control-SD, *n* = 9 at P12, *n* = 8 at P13, *n* = 6 at P15). **(B)** The latency to onset of spasms did not differ significantly between MAM and control groups treated with a single NMDA dose. **(C)** MAM-exposed rats exhibited significantly greater numbers of spasms in response to repeated NMDA administration on P12, P13, and P15 (MAM-MD, *n* = 15; 65.3 ± 6.2 at P12, *p* = 0.003; 80.1 ± 7.2 at P13, *p* = 0.001; 74.5 ± 10.1 at P15, *p* = 0.002) compared to prenatally saline-exposed rats (control-MD, *n* = 9, 35.1 ± 5.9 at P12, 31.7 ± 3.2 at P13, 29.6 ± 6.0 at P15). The number of spasms differed significantly between the control and the MAM groups over time (*p* < 0.001). **(D)** The latency to onset of spasms was significantly shorter in MAM rats than in controls with a significant group × time interaction (*p* = 0.032). The mean latency to onset of spasms in the MAM-MD group at P13 (1,313.7 ± 57.6 s) was significantly shorter than that in the control-MD group (1,543.8 ± 78.3 s, *p* = 0.03). **(E)** In MAM-SD rats, vigabatrin (VGB) significantly reduced the number of spasms at 125 mg/kg (VGB125, *n* = 11, 8.8 ± 2.6, *p* < 0.001) and 250 mg/kg (VGB250, *n* = 8, 4.3 ± 1.0, *p* < 0.001) compared to VGB-naïve MAM group rats (*n* = 10, 42.7 ± 6.4). **(F)** Pretreatment with VGB125 and VGB250 significantly prolonged the mean latency to onset of tailing (VGB125, 1,404.8 ± 70.5 s, *p* = 0.01; VGB250, 1,660.4 ± 113.8 s, *p* = 0.001) and first full spasms (VGB125, 2,047.8 ± 153.5 s, *p* = 0.012; VGB250, 2,246.9 ± 398.3 s, *p* = 0.016) compared to VGB-naïve MAM group rats (tailing, 1,091.5 ± 80.7 s; first full spasm, 1,480.9 ± 82.1 s) dose-dependently. In contrast, adrenocorticotropic hormone (ACTH, *n* = 6) had little effect on the number of spasms and the latency to onset of spasms. Values expressed as mean ± SEM. *p < 0.05 using Mann–Whitney *U* test for comparison between the two groups. ^†^*p* < 0.05 using repeated measure ANOVA for main effect of group (between group) on the number of spasms or the latency to onset of spasms. ^‡^*p* < 0.05 using repeated measure ANOVA for interaction effect of time-by-group on the number of spasms or the latency to onset of spasms.

After NMDA injection, the rats were motionless for 10 min and subsequently became hyperactive and displayed frequent tail twisting (tailing) followed by moving around in circles. Eventually, the rat developed spasms manifesting clusters of hyperflexion of whole body, which are very similar to the human condition and a previous animal model (19, 22). The number of full spasms triggered by a single NMDA dose was significantly higher in MAM-exposed rats (MAM-SD) compared to age-matched controls (control-SD) at P12 (65.3 ± 6.2 vs. 35.1 ± 5.9, *n* = 9 vs. *n* = 15; *p* = 0.003) and P13 (18.8 ± 3.8 vs. 4.4 ± 1.2, *n* = 8 in both groups; *p* = 0.002) but not P15 (42.7 ± 6.4 vs. 39.8 ± 4.8, *n* = 6 vs. *n* = 10; *p* = 0.958) (Figure 4A). The MAM-SD rats also demonstrated significantly shorter latency to tailing onset at P13 than controls-SD rats (966.6 ± 49.6 vs. 1,532.6 ± 124.5 s, *p* < 0.001), and the latency to onset of full spasms was not significantly different (Figure 4B). In experiments using multiple NMDA administrations, a significantly greater number of full spasms was observed following each administration in the MAM group (MAM-MD) compared to controls (control-MD) at P12 (65.3 ± 6.2 vs. 35.1 ± 5.9; *p* = 0.003), P13 (80.1 ± 7.2 vs. 31.7 ± 3.2, *p* = 0.001), and P15 (74.5 ± 10.1 vs. 29.6 ± 6.0, *p* = 0.002) (Figure 4C). The latency to onset of full spasms following exposure to multiple NMDA doses also differed significantly between control-MD and MAM-MD rats with a significant group × time interaction (interaction *p* = 0.032) (Figure 4D). The mean latency to onset of full spasms in MAM-MD rats was significantly shorter compared to controls at P13 (1313.7 ± 57.6 vs. 1543.8 ± 78.3 s, *p* = 0.03).

Acute pretreatment with 125 mg/kg vigabatrin (VGB125, *n* = 11) or 250 mg/kg (VGB250, *n* = 8) significantly reduced the number of spasms induced by a single NMDA dose at P15 in MAM-exposed rats compared to VGB-naïve MAM-exposed rats (VGB125, 8.8 ± 2.6 vs. 42.7 ± 6.4, *p* < 0.001; VGB250 4.3 ± 1.0, *p* < 0.001; *n* = 10 drug-naïve rats) (Figure 4E). In addition, acute pretreatment with VGB125 or VGB250 significantly prolonged the mean latency to onset of tailing compared to controls (VGB125, 1,404.8 ± 70.5 vs. 1,091.5 ± 80.7 s, *p* = 0.01; VGB250, 1,660.4 ± 113.8 s, *p* = 0.001) and delayed the onset of the full spasm (VGB125, 2,047.8 ± 153.5 vs. 1,480.9 ± 82.1 s, *p* = 0.012; VGB250, 2,246.9 ± 398.3 s, *p* = 0.016) (Figure 4F). In contrast, acute pretreatment with ACTH had little effect on the number of spasms and the latency to onset of spasms.

### Stronger FOs in MAM-Exposed Infant Rats

**Figure 5:**
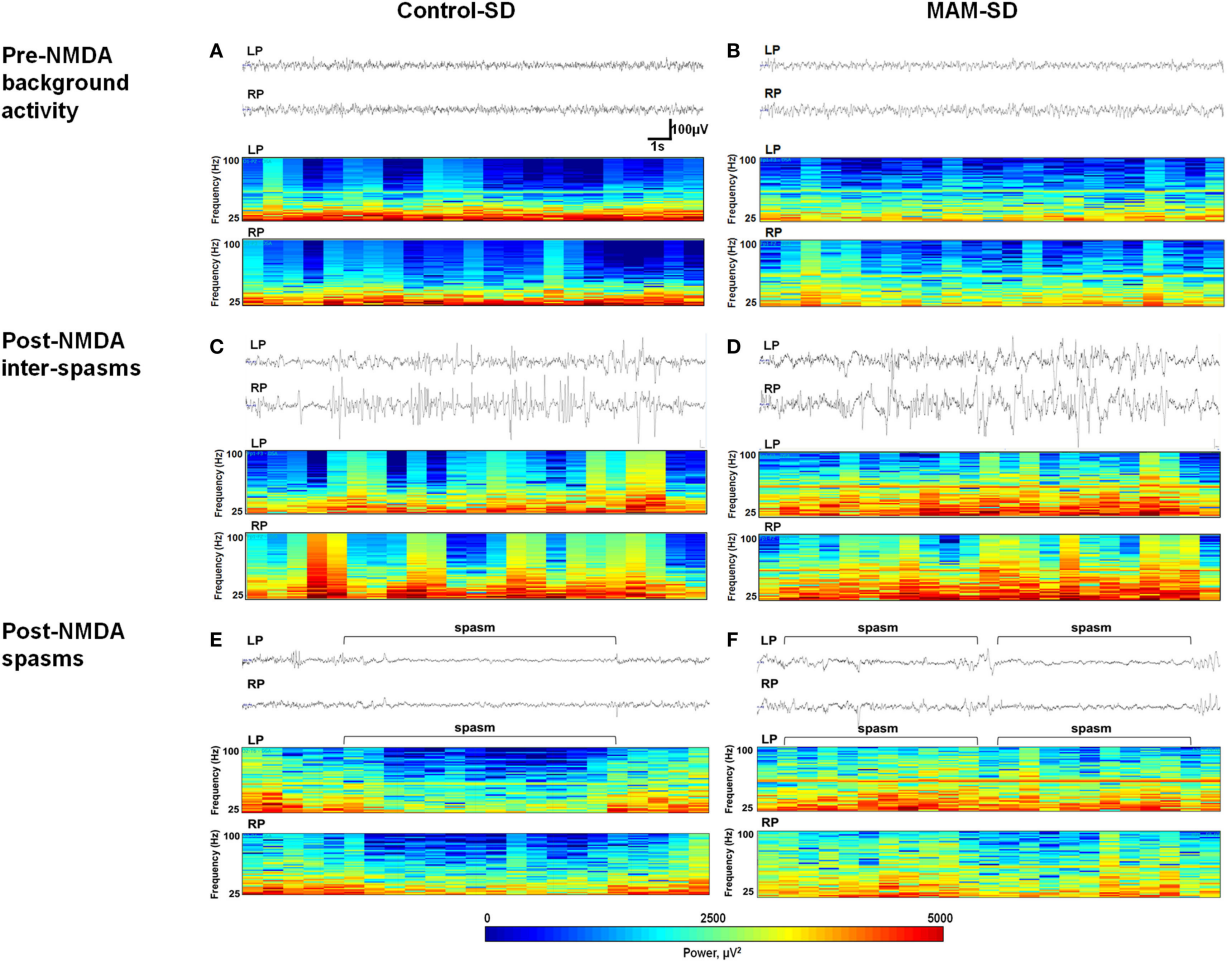
Electroencephalographic (EEG) recordings and color density spectral arrays (CDSAs) of *N*-methyl-d-aspartate (NMDA)-induced spasms and inter-spasm periods. Monopolar EEG recordings were performed from two intracranial electrodes on the left parietal (LP) and right parietal (RP) areas. Upper panels present 30-s EEG samples from methylazoxymethanol acetate (MAM) and control group animals and the lower panels the corresponding CDSAs for same period bandpass filtered at 0.5–100 Hz. The CDSAs display a color-coded, three-dimensional contour plot with time on the horizontal axis, frequency (25–100 Hz) on the vertical axis and color scale power (μV^2^) [see scale below lower panel of **(E,F)**]. **(C,D)** Both prenatally MAM and control rats administered a single NMDA dose (SD) showed frequent spikes intermixed with high-amplitude slow wave activity. The corresponding CDSAs demonstrate more prominent fast oscillations (25–100 Hz) during post-NMDA inter-spasms periods than during pre-NMDA resting background activity **(A,B)**. **(E,F)** Sample EEGs during post-NMDA spasms indicate low-amplitude fast activities superimposed on the attenuated background in both control-SD and the MAM-SD groups. In the corresponding CDSAs, more prominent FOs were revealed in the MAM-SD group compared to the control-SD group.

Representative EEG recordings (upper panel) and corresponding CDSAs (lower panel) of pre-NMDA resting periods and post-NMDA spasms and inter-spasms periods are presented in Figure 5. After NMDA administration, both MAM and control rats treated with a single NMDA dose exhibited frequent spikes intermixed with high amplitude slow wave activity during inter-spasms periods. The corresponding CDSAs revealed more noticeable FOs in the 25–100 Hz range during post-NMDA inter-spasms period compared to pre-NMDA background activity (Figures 5C,D). The EEGs during single-dose NMDA-induced spasms showed low-amplitude fast activities in both groups, while the corresponding CDSAs in the MAM group revealed more prominent FOs in the 25–100 Hz range (Figures 5E,F).

**Figure 6:**
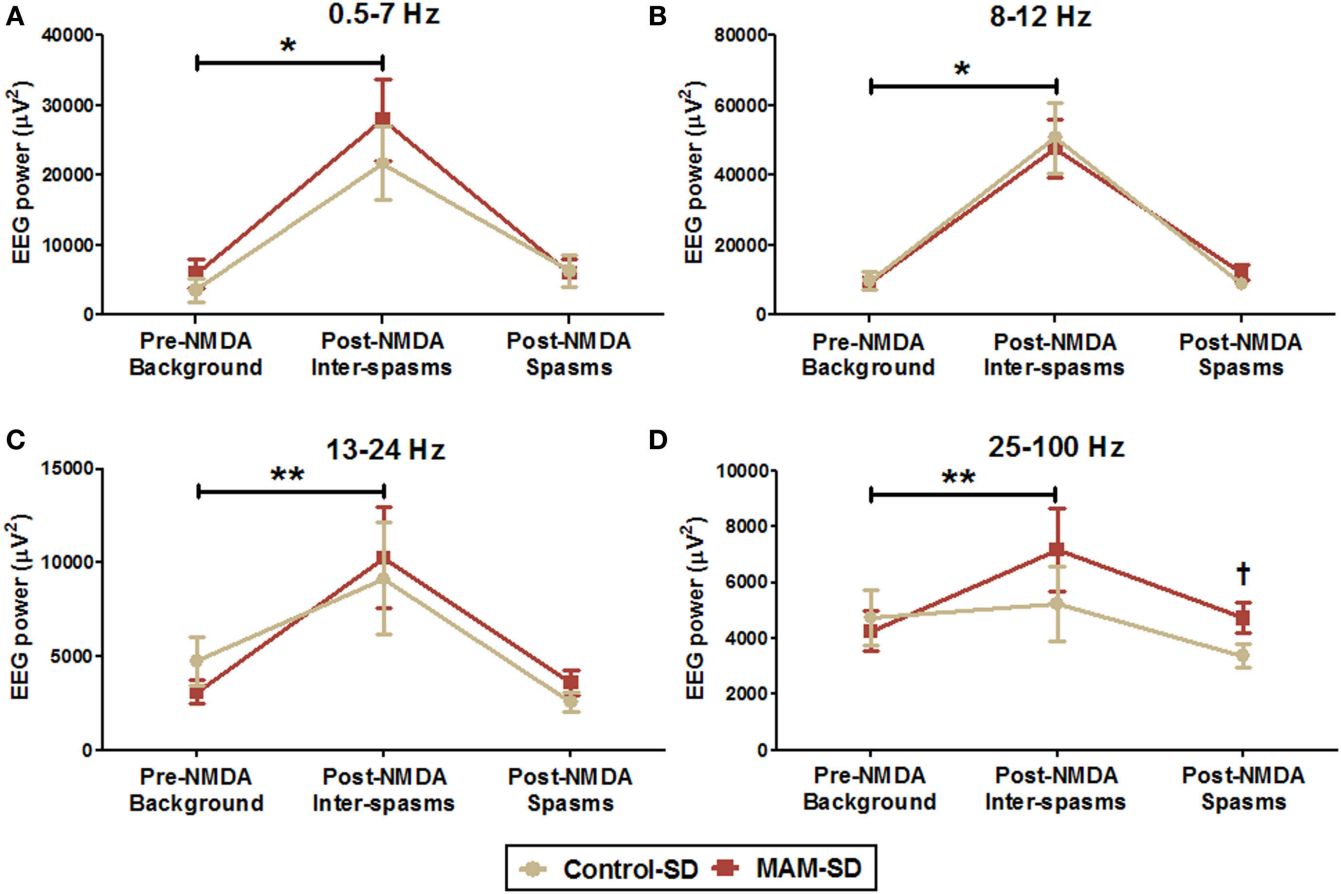
Quantitative spectral power analysis of electroencephalographies (EEGs) from methylazoxymethanol acetate (MAM)-exposed (*n* = 9) and saline-exposed rats (*n* = 9) before and after administration of a single *N*-methyl-d-aspartate (NMDA) dose (SD). **(A,B)** Mean EEG powers (μV^2^) of delta and theta (0.5–7 Hz) and alpha (8–12 Hz) frequency bands were significantly higher in the post-NMDA inter-spasms period than during the pre-NMDA background period for both groups. **(C,D)** The MAM-SD group exhibited higher EEG powers at the beta (13–24 Hz) and fast oscillation (FO; 25–100 Hz) bands during post-NMDA inter-spasms period than during the pre-NMDA period, while the control-SD group did not. **(D)** The EEG power of the FO band during spasms events was significantly higher in the MAM-SD group (4,721.9 ± 544.2 µV^2^) than in the control-SD group (3,374.0 ± 409.2 µV^2^, *p* = 0.047). Values expressed as mean ± SEM. *p < 0.05 using the Mann–Whitney *U* test for comparison between the pre-NMDA background activity and the post-NMDA inter-spasms in both two groups. **p < 0.05 using the Mann–Whitney *U* test for comparison between pre-NMDA background activity and post-NMDA inter-spasms in the MAM-SD group. ^†^*p* < 0.05 using the Mann–Whitney *U* test for comparison between the groups.

Quantitative measure of power spectra derived from these EEGs were compared between control-SD (*n* = 9) and the MAM-SD rats (*n* = 9) (Figure 6). Before NMDA administration, there were no significant differences in EEG band powers between groups. Both groups showed significantly higher EEG powers (μV^2^) at delta and theta (0.5–7 Hz) and alpha (8–12 Hz) frequency bands during post-NMDA inter-spasms periods than during pre-NMDA periods (controls at 0.5–7 Hz: 3,535.7 ± 1,642.9 vs. 21,637.2 ± 5,235.5 µV^2^, *p* = 0.001; MAMs at 0.5–7 Hz: 5,879.7 ± 2,112.3 vs. 27,841.4 ± 5,784.0 µV^2^, *p* < 0.001; controls at 8–12 Hz: 9,855.8 ± 2,666.9 vs. 50,805.1 ± 10,103.5 µV^2^, *p* < 0.001; MAMs at 8–12 Hz: 9,119.2 ± 1,804.8 vs. 47,678.1 ± 8,162.4 µV^2^, *p* < 0.001) (Figures 6A,B). However, only the MAM group showed a significant increase in beta power (13–24 Hz) and FO (25–100 Hz) power during post-NMDA inter-spasms periods (13–24 Hz: 10,247.47 ± 2,693.17 vs. 3,108.16 ± 662.30 µV^2^, *p* < 0.001; 25–100 Hz: 7,158.45 ± 1,469.59 vs. 4,246.30 ± 717.46 µV^2^, *p* = 0.005) than pre-NMDA periods (Figures 6C,D). During post-NMDA spasms, MAM-SD rats showed significantly higher FO power than control-SD rats (5,264.1 ± 350.7 vs. 3,994.8 ± 327.2 µV^2^, *p* = 0.029) (Figure 6D).

## Discussion

Cortical malformations are common etiologies of refractory epilepsy, and epileptic spasms are frequently associated with this pathological condition (23). Here, we characterized MAM-induced MCDs in young rats *in vivo* and established a new infant rat model of epileptic spasms based on the combination of chemically induced MCDs and postnatal NMDA administration.

The MAM treatment protocol could reproduce the anatomical disruptions observed in previous studies of adult rats (8, 24) in infant rats, which was confirmed by *in vivo* MRI. Open-field behavior and fear-associated learning and memory were also altered in this animal model at younger ages than previously shown (25, 26). The reduced ambulation speed in the central zone of the open field can be interpreted as reduced anxiety as rats with normal anxiety prefer to stay in the periphery (27). The fear responses are associated with hippocampus- and amygdala-dependent emotional learning and memory (28) and the altered anxiety/fear-associated memory in this MCD model may be related to pathological changes in the hippocampus and amygdala (29). In contrast, multiple bouts of NMDA-induced spasms in this study did not have a significant impact on these behavioral parameters (data not shown) suggesting that the underlying MCDs are the primary factor leading to cognitive impairment in accordance with a previous study (26).

Based on the well-characterized MAM model of MCDs and a previously reported model of epileptic spasms (19), we tested the susceptibility of MCD rats to NMDA-provoked epileptic spasms. MAM-exposed infant rats exhibited increased susceptibility to spasms triggered by both single and multiple doses of NMDA with larger (higher power) FOs during spasms and inter-spasms periods compared to controls. Based on intra-operative recordings of patients with cortical malformation-associated epilepsy, it is well established that malformed brain regions are potential sites of seizure generation with dense FOs (30). Studies of MAM-exposed rats also indicated a lower threshold for generation of epileptiform activity (7, 11, 12). This higher seizure propensity may explain the increased number of spasms during infancy in our model. The age-specific difference in spasm susceptibility observed in MAM-exposed rats following a single NMDA dose also suggests a parallel age-dependent change in neuronal or network excitability. Indeed, susceptibility to seizures changes over the postnatal development with the development of neural circuits (31, 32). The immature MCD brain may exhibit a time window of over-excitation during development, which appeared to be at P12–P13 in our model based on peak NMDA response. In addition, earlier spasms in prenatally MAM-exposed rats increased subsequent spasms susceptibility as previously reported in prenatal betamethasone-exposed rats (22) suggesting that post-spasm neuronal network changes further lower spasms threshold.

Fast oscillations (>50 Hz) during epileptic spasms are generated by the neocortex and could be a biomarker of the ictal focus (33). Previous studies also reported the presence of abnormally increased FOs in ictal and interictal periods with epileptic spasms in human patients and animal models (33, 34). In the present study, we found that rats with MCDs showed higher power FOs (25–100 Hz) during spasms and inter-spasms periods compared to controls, which may reflect increased epileptogenicity of the malformed cortex.

The efficacies of ACTH and VGB, which are treatments of choice for the human condition (35), were also tested against NMDA-induced spasms in this model. While acute ACTH pretreatment had no impact on NMDA-induced spasms, acute VGB pretreatment significantly suppressed their development consistent with previous studies in other animal models of symptomatic epileptic spasms (18). Considering the good therapeutic effects of acute pretreatment with ACTH on the suppression of spasms in animal models of cryptogenic epileptic spasms (19), it is possible that different underlying etiology of spasms may explain the different responses to ACTH. On the other hand, a deficit in GABAergic inhibition found in animal models of MCDs (11, 12, 36) could account for the good response to VGB. Recent evidence suggests that changes in GABAergic transmission contribute to the abnormal excitability in cortical dysplasia (37). The disruption of GABAergic system in seizures associated with cortical malformation is also supported by the particular efficacy of the GABA transaminase inhibitor VGB against epileptic spasms in tuberous sclerosis complex (35), which shares certain pathophysiological traits with MCDs (38). Thus, the VGB sensitivity of this new model of epileptic spasms validates again its analogy to human epileptic spasms due to MCDs and suggests the putative mechanisms involved in epileptogenesis of epileptic spasms associated with MCDs.

A significant advantage of this model over spontaneous seizure models is that we can trigger spasms at specific times for examination during the rat infancy. This model also has structural and electrophysiological features of MCDs-associated epileptic spasms in humans. Although two-channel EEG data are not sufficient to document the disorganization and multifocality of hypsarrhythmia, high amplitude fast activities similar to the previous report with NMDA-induced spasms (19) are shown and the difference between the two models with different structures are analyzed. In future studies with this model, alterations of cytoarchitectures and neurometabolites should be investigated to find the pathomechanism of spasms susceptibility in MCDs, which will enable the development of new targeted therapy. In addition, this model can be used as the preclinical tests for the new antiepileptic drugs.

In conclusion, the present study provides an important validation of a new model of epileptic spasms with underlying MCDs pathology. MCDs were confirmed in young rats by *in vivo* MRI. Postnatal NMDA administration produced more intense spasms with stronger FOs compared to controls. The efficacy of VGB for suppression of spasms in this rat model was comparable to that against human epileptic spasms with structural etiologies. Therefore, this model may provide further insights into the pathogenesis, progression, and clinical features of epileptic encephalopathies associated with MCDs and hold great promise as a substrate to identify novel therapeutic targets for this devastating disorder.

## Ethics Statement

All experiments were approved by the Institutional Animal Care and Use Committee of the Ulsan University College of Medicine and conducted in accordance with the Revised Guide for the Care and Use of Laboratory Animals [NIH GUIDE, 25(28), 1996].

## Author Contributions

E-HK, M-SY, and T-SK were involved in the conception and design of the study. E-HK, M-SY, ML, E-JK, and W-HS were involved in data acquisition and data interpretation for the study. E-HK and M-SY were involved in drafting and editing the manuscript and the figures. M-SY and T-SK reviewed submitted version of manuscript and supervised the study.

## Conflict of Interest Statement

The authors declare that the research was conducted in the absence of any commercial or financial relationships that could be construed as a potential conflict of interest.
